# Hemorrhagic Fever with Renal Syndrome, Russia

**DOI:** 10.3201/eid2512.181649

**Published:** 2019-12

**Authors:** Evgeniy A. Tkachenko, Aydar A. Ishmukhametov, Tamara K. Dzagurova, Alla D. Bernshtein, Viacheslav G. Morozov, Alexandra A. Siniugina, Svetlana S. Kurashova, Alexandra S. Balkina, Petr E. Tkachenko, Detlev H. Kruger, Boris Klempa

**Affiliations:** Russian Academy of Sciences, Moscow, Russia (E.A. Tkachenko, A.A. Ishmukhametov, T.K. Dzagurova, A.D. Bernshtein, A.A. Siniugina, S.S. Kurashova, A.S. Balkina);; Sechenov First Moscow State Medical University, Moscow (E.A. Tkachenko, A.A. Ishmukhametov, P.E. Tkachenko); Gepatolog, LLC, Samara, Russia (V.G. Morozov);; Institute of Virology, Helmut-Ruska-Haus, Charité Medical School, Berlin, Germany (D.H. Kruger, B. Klempa);; Biomedical Research Center, Slovak Academy of Sciences, Bratislava, Slovakia (B. Klempa)

**Keywords:** hantavirus, hemorrhagic fever with renal syndrome, epidemiology, viruses, Russia, zoonoses

## Abstract

In Russia, 131,590 cases of hemorrhagic fever with renal syndrome caused by 6 different hantaviruses were reported during 2000–2017. Most cases, 98.4%, were reported in western Russia. The average case-fatality rate was 0.4%, and strong regional differences were seen, depending on the predominant virus type.

Hemorrhagic fever with renal syndrome (HFRS) is caused by hantaviruses (order *Bunyavirales*, family *Hantaviridae*), enveloped, single-strand, negative-sense RNA viruses, predominantly carried by rodents and insectivores. In Asia, the primary HFRS pathogens are Hantaan virus (HTNV), Amur virus (AMRV), and Seoul virus (SEOV); in Europe, the primary pathogens are Puumala virus (PUUV) and Dobrava-Belgrade virus (DOBV) (*1*). 

Russia, bordered by Europe in the west and Asia in the east, included HFRS in the official reporting system of the Ministry of Public Health in 1978 (*2*). Clinical and laboratory diagnoses for reported cases are confirmed serologically by indirect immunofluorescence assay (Diagnostikum HFRS; Federal Scientific Center for Research and Development of Immune and Biological Products of the Russian Academy of Sciences, http://chumakovs.ru). 

HFRS has the highest incidence rate of all reportable zoonotic viral diseases in Russia. In the west, in administrative regions close to the border with Europe, reported cases mainly are caused by PUUV carried by bank voles (*Myodes glareolus*) and to a lesser extent by 2 types of DOBV, Kurkino virus (KURV) and Sochi virus (SOCV) (*3*). Vectors for DOBV subtypes in western Russia are the western subtype of striped field mouse (*Apodemus agrarius agrarius*), which hosts KURV, in the central regions; and the Black Sea field mouse (*A. ponticus*), which hosts SOCV, in southern regions. In eastern Russia, near the border with Asia, HFRS cases primarily are caused by HTNV carried by the eastern subtype of striped field mouse (*A. agrarius mantchuricus*), AMRV carried by the Korean field mouse (*A. peninsulae*), and, less frequently, SEOV carried by the Norway rat (*Rattus norvegicus*) (*4*,*5*).

During 2000–2017, a total of 68 of Russia’s 85 administrative regions reported 131,590 HFRS cases, an annual average rate of 4.9 cases/100,000 inhabitants (Figure, panel A). Annual incidence rates varied greatly, and epidemics occurred every 2–4 years with occasional 2-year peaks, such as in 2008–2009 and 2014–2015. This phenomenon is related to sequential independent epidemic years in 2 distinct, highly affected regions rather than geographically synchronized hantavirus activity on a nationwide scale.

**Figure:**
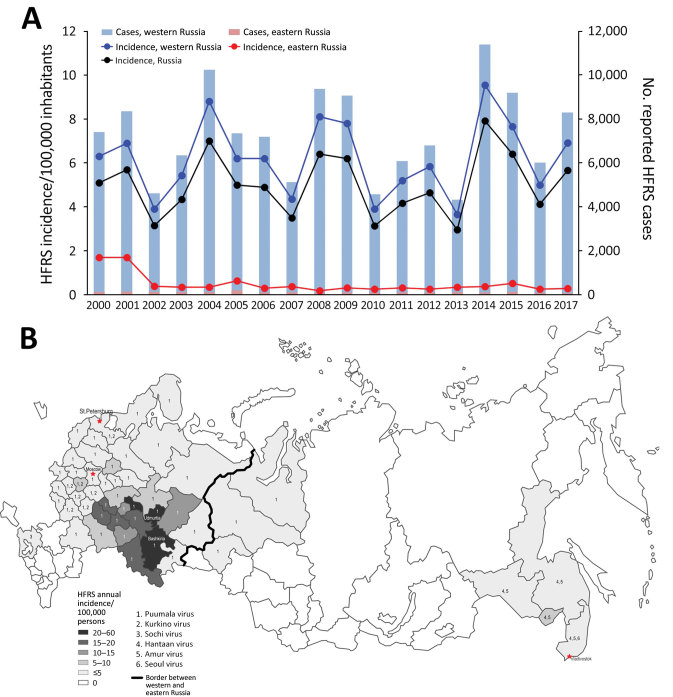
Distribution of hemorrhagic fever with renal syndrome caused by hantavirus in Russia, 2000–2017. A) Mean number of reported cases and incidence of disease, by region; B) geographic distribution and incidence rate of causative agents (indicated by numbers). Red stars indicate primary cities in Russia.

HFRS cases were distributed unevenly throughout Russia. Western Russia reported 129,530 (98.4%) cases in 52/60 regions and an average annual incidence of 6.0 cases/100,000 persons. Eastern Russia reported only 2,060 (1.6%) cases in 16/25 regions and an average annual incidence of 0.4 cases/100,000 persons (*2*). The Ural and Ural-Volga-Viatka foothill areas, which encompass 11 administrative regions of western Russia, had the highest HFRS incidence rates, >10 cases/100,000 persons (Figure, panel B). Overall, 77% of HFRS cases in Russia were reported from these 11 regions, which are characterized by lime forests that provide suitable habitat for the bank vole, the reservoir host of PUUV. Among these regions, 2 had the highest incidence rates in the country: Udmurtia had 61.4 cases/100,000 persons and Bashkiria 47.5 cases/100,000 persons.

In eastern Russia, the 4 administrative regions closest to Asia reported HFRS cases. Vladivostok reported 1,089 cases and an incidence rate of 3.0 cases/100,000 persons; Khabarovsk reported 519 cases and an incidence rate of 2.1 cases/100,000 persons; Amur reported 71 cases and an incidence rate of 0.4 cases/100,000 persons; and Jewish Autonomous Region reported 189 cases and an incidence rate of 5.8 cases/100,000 persons. Siberia reported only 179 cases, mainly from western Siberia, which likely were imported cases in temporary oil and gas field workers from other hantavirus-endemic regions, such as the neighboring Udmurtia and Bashkiria.

During 2000–2017, Russia had 564 fatal cases of HFRS, 483 in the east and 81 in the west. The overall case-fatality rate was 0.4%, but rates varied by region. Central regions of western Russia had case-fatality rates of 0.3%, but the Black Sea coastal area of western Russia, where highly pathogenic SOCV occurs, had a 14% HFRS case-fatality rate. The far eastern regions, which have endemic highly pathogenic HTNV, had a 7% case-fatality rate (*6**–**9*).

HFRS appears to affect persons 20–50 years of age most frequently (65%), and ≈80% of cases in Russia were in men. Only 3,157 (2.4%) cases were reported among children <14 years of age. Most HFRS cases in western Russia occurred during the summer and autumn, but cases in the far eastern part of the country occurred in autumn and winter (*4*,*5*).

Comparative analyses of clinical courses indicated that even though infections by all recognized causative agents can cause mild, moderate, and severe clinical forms of HFRS, the frequency differs depending on the causative agent. SOCV infections had greater incidence of severe HRFS and high case-fatality rates (14%) and HTNV infections had case-fatality rates of 5%–8%, whereas PUUV, SEOV, and KURV infections had case-fatality rates <1% (*8*–*10*). Of note, 97.7% of HFRS cases in Russia are reportedly caused by PUUV (*5*), possibly explaining the overall low case-fatality rate in the country. Nevertheless, considering the high case numbers reported from the west, HFRS remains a public health threat in Russia.
